# Good problems to have? Policy and societal implications of a disease-modifying therapy for presymptomatic late-onset Alzheimer’s disease

**DOI:** 10.1186/s40504-020-00106-2

**Published:** 2020-10-12

**Authors:** Misha Angrist, Anna Yang, Boris Kantor, Ornit Chiba-Falek

**Affiliations:** 1 Initiative for Science and Society and Social Science Research Institute, Duke University, Durham, North Carolina 27708-0222 USA; 2 Duke University, Durham, USA; 3 Duke University Department of Neurobiology, Durham, North Carolina 27710-3209 USA; 4 Duke University Department of Neurology, 311 Research Drive, Durham, North Carolina 27710-2900 USA; 5 Duke Center For Genomic And Computational Biology, Durham, USA

**Keywords:** Alzheimer’s, Therapy, Access, Diagnosis, Gene editing, Service delivery, Infrastructure, Drug pricing

## Abstract

In the United States alone, the prevalence of AD is expected to more than double from six million people in 2019 to nearly 14 million people in 2050. Meanwhile, the track record for developing treatments for AD has been marked by decades of failure. But recent progress in genetics, neuroscience and gene editing suggest that effective treatments could be on the horizon. The arrival of such treatments would have profound implications for the way we diagnose, triage, study, and allocate resources to Alzheimer’s patients. Because the disease is not rare and because it strikes late in life, the development of therapies that are expensive and efficacious but less than cures, will pose particular challenges to healthcare infrastructure. We have a window of time during which we can begin to anticipate just, equitable and salutary ways to accommodate a disease-modifying therapy Alzheimer’s disease. Here we consider the implications for caregivers, clinicians, researchers, and the US healthcare system of the availability of an expensive, presymptomatic treatment for a common late-onset neurodegenerative disease for which diagnosis can be difficult.

## Introduction

As the sixth leading cause of death in the United States, Alzheimer’s disease (AD) constitutes a major public health crisis. Nearly six million Americans over the age of 65 are living with AD; by 2050 that number is expected to approach 14 million (Alzheimer's Association 2020). The total lifetime cost of caring for an AD patient is estimated to be on the order of $350,000; in 2018, the financial burden of physical and emotional caregiving for AD (and other dementia) patients approached $12 billion (El-Hayek et al. 2019).

Meanwhile, the AD drug development pipeline has been marked by decades of failure. The biology of AD is complex—whether one develops late-onset AD (LOAD), for example, appears to be a function of one’s genetic risk at many loci, family history, gender, and environmental factors such as diet, educational attainment, and physical and intellectual activity (Dufouil and Glymour 2018; van der Lee et al. 2018; Hersi et al. 2017), modulation of which can help delay and/or prevent LOAD onset (Ko and Chye 2020). In light of this complexity, the longstanding idea that the relationship between accumulation of beta-amyloid and AD pathogenesis is a straightforward one has become increasingly tenuous (Begley 2019; Huang et al. 2019; Ricciarelli and Fedele 2017).

While AD remains a daunting biological and clinical problem, recently, a number of other devastating diseases long thought to be intractable—including spinal muscular atrophy (Al-Zaidy et al. 2019), transfusion-dependent β-thalassemia (Thompson et al. 2018), hemophilia A (Garde 2019), and biallelic RPE65-mediated inherited retinal disease (Voretigene neparvovec-rzyl (Luxturna) for inherited retinal dystrophy 2018)—have begun to be treated with somatic-cell and gene therapies (albeit very expensive ones—more on that below). While similar treatments for dementia are still a significant distance from commercialization, there are hints that viral vector-mediated gene therapy (introduction of exogenous DNA or RNA) and/or gene editing (correction of endogenous gene [s]) might be used to treat patients LOAD within the next few years (Bustos et al. 2017; Cota-Coronado et al. 2019; Giau et al. 2018; Prendecki et al. 2020; Raikwar et al. 2018; Iqubal et al. 2020). Moreover, insights into AD pathology have come from surprising and enlightening places; for example, recent evidence suggests that the tyrosine kinase inhibitor and leukemia treatment nilotinib has salutary effects on AD biomarkers (Turner et al. 2020).

The scientific challenges, feasibility and exact timeline of an AD treatment breakthrough are all well beyond the scope of this commentary, which is instead aimed at what happens when we eventually reach the finish line (or, more accurately, the first of many finish lines). Our thought experiment is to imagine a world in which gene-editing technology could begin to deliver us from the current cycle of futility. What happens when science is finally able to harness gene editing (or another similarly powerful technique) in the service of a disease-modifying therapy (DMT) for Alzheimer’s disease? Presumably the boon to human health would be enormous: millions of AD patients would suffer less and fewer loved ones and caregivers would have to watch that suffering, to say nothing of the untold hours and dollars they would no longer have to spend trying to mitigate it.

In 2017 a group of British experts on neurodegenerative disease reported on a consensus meeting designed to articulate the ways in which the United Kingdom’s current services for dementia patients would have to change in order to accommodate the availability of a DMT for AD patients, especially presymptomatic and prodromal cases (Ritchie et al. 2017). Others have considered ethical issues specific to treatment of late-stage AD patients (Watt et al. 2019). Here we take a broader view of that prospect and, from a fairly high altitude, consider some of the logistical, ethical, policy and financial implications of a DMT aimed at presymptomatic and prodromal AD patients.

## A DMT for whom?

At present a definitive diagnosis of AD is only possible by identifying telltale brain lesions (amyloid plaques and neurofibrillary tangles) at autopsy (Khan and Alkon 2015). This makes both the development and delivery of a DMT to AD patients exceedingly difficult and likely explains in no small part why the drug development pipeline has yet to bear substantive fruit. Without the ability to precisely and reliably distinguish current and future AD patients from patients with other forms of dementia stemming from, say, cerebrovascular disease, Lewy body disease, or frontotemporal lobar degeneration (Alzheimer's Association 2020), deciding whom to include in experimental and control arms of any prospective clinical trial remains fraught (indeed, even the hallmark tau protein pathology is strikingly heterogeneous among LOAD patients (Dujardin et al. 2020)). At the other end of the pipeline, there are equally profound consequences—both clinical and financial—of giving a DMT to a patient who doesn’t truly (and perhaps never will) have AD (false positive). By the same token, if, because of imprecise diagnostics, we overlook a patient who truly *does* have (or goes on to have) AD but we don’t discover that fact until autopsy (false negative), then we will have missed an opportunity to help that patient and her caregivers.

The good news is that both diagnostic acumen and assessment of risk are improving. In results from the ongoing, prospective, community-based Rotterdam study, for example, investigators showed that by combining assessments of parental history of AD along with APOE genotype (the major genetic risk factor for AD) and a genetic risk score aggregating 23 other genetic variants associated with the disease, they could identify a subset of individuals whose risk for developing AD by age 85 was greater than 90 percent (Dufouil and Glymour 2018; van der Lee et al. 2018). Other groups have recently presented data indicating that a sensitive, specific, blood-based diagnostic is possible (Marchione 2019). These results suggest that, although screening out false positives and false negatives remains challenging, we might stratify future presumptive AD patients for DMT trials based on Bayesian risk estimates, that is, a more sophisticated version of what’s been done in the past (Geifman et al. 2018; Hampel et al. 2018). Cost notwithstanding, even greater precision might be possible by including data from imaging, additional biomarkers and cognitive testing (Khan and Alkon 2015; Lange et al. 2018; Riedel et al. 2018; Keshavan 2019).

It appears increasingly likely that, in many people, greater physical activity can reduce beta-amyloid burden, neurodegeneration and cognitive decline in healthy aging people (Rabin et al. 2019). Thus, moving forward, incorporating complex lifestyle data and an array of biomarkers is apt to be a challenging but critical step in the stratification of DMT trial participants and, eventually, potentially eligible patients (Lombardi et al. 2020).

While recent work in cell reprogramming and repair has shown some promise (Barker et al. 2018), any DMT for AD is likely to be of the greatest benefit to presymptomatic and perhaps prodromal patients (Ritchie et al. 2017). This makes both biological sense (it is easier to preserve than to restore neuronal function) and practical sense (successfully treated earlier-stage patients are likely to experience better quality of life). Consequently, accuracy considerations notwithstanding, the availability of a DMT for AD will cause the burden on the diagnostic enterprise to grow dramatically—CSF and plasma biomarker assays, brain imaging, cognitive testing, and memory clinics are all expensive undertakings. And for its part, the US is already short on both geriatricians (Pitkälä et al. 2018) and neurologists (Dall et al. 2013).

At the same time, a DMT for presymptomatic/prodromal AD patients means that patients with more advanced dementia and persons whose risk scores fail to meet the threshold for study inclusion and/or treatment but still progress to AD, are at risk of being left behind. One instructive precedent is the apparent divide between some early-stage and metastatic breast cancer patients and activists, as fundraising and research efforts have emphasized the needs of the former, much to the consternation of the latter (Corneliussen-James 2015). How will the AD community respond to patients who are not able to benefit from a DMT? Will such patients avail themselves of it anyway and, if so, to what end and at what cost?

## What do we mean by “disease-modifying?” and how much are we willing to pay?

Given that the most responsive treatment-eligible populations are likely to be presymptomatic or prodromal, the question then becomes how to measure success in treated patients. Is it patients simply never succumbing to AD by a certain age? Is it them dying of something else after a certain age? (Given the disproportionate mortality among older people with COVID-19, the coronavirus pandemic is likely to have an outsized effect on AD patients (Brown et al. 2020).) And what if “something else” is another form of dementia?

As mentioned, the FDA-approved AD pharmacopoeia is nearly bare and so one is tempted to dismiss these questions as good problems to have. But they pose clinical, scientific and economic challenges that, in aggregate, force us to consider what AD patients and their caregivers might get for what are likely to be massive expenditures. As we begin to see gene therapies become available for other deadly diseases, including some neurodegenerative ones, we are also learning that while they may be hailed in the media as “miracles,” the reality is often more complicated. A recent analysis of Luxturna (voretigene neparvovec-rzyl) for the treatment of a rare retinal dystrophy (RPE65-mediated blindness), for example, looked at FDA review documents written prior to the drug’s December 2017 approval. The author noted that the drug, which costs $850,000 ($425,000 per eye), is “well short of the cure that patients desire,” even in the most responsive patients, despite numerous media claims to the contrary (Darrow 2019).

So what does this cautionary tale have to do with a hypothetical DMT for AD? If a drug meant to treat a rare disease commands an exorbitant price and fails to deliver what its maker has promised, it is certain to be an injustice to patients and their caregivers and perhaps a nontrivial burden on the healthcare system writ large. Because AD is not rare, the consequences of approving and selling a drug meant to treat it that does not substantially improve quality of life or mitigate both financial expenditures and caregiver burdens would likely be orders of magnitude more catastrophic for the US healthcare system and our collective trust in it. By the same token, the giant market commanded by patients suffering from a common disease will presumably generate a more vibrant, competitive market, assuming the biology can be brought to heel and multiple treatments emerge.

This brings us back to the initial question: what do we mean by “disease-modifying?” In other words, where exactly *are* we setting the bar? A partial counterexample to the Luxturna might be the newest class of drugs used to treat hepatitis C, which, like LOAD, was not rare (as of 2016^1^). When the initial treatment, Sovaldi (sofosbuvir), entered the US market at a price of $84,000 for a 12-week course of treatment, Congress and patient advocates responded with howls of protest (Cohen 2013; Loftus 2014). More than 5 years on, direct-acting antivirals like Sovaldi have yielded a cure rate for hepatitis C in excess of 90 percent (Baumert et al. 2019). And while pricing and access to these expensive drugs in the developing world vary widely, some promising ways forward have begun to emerge (Douglass et al. 2018).

Direct-acting antivirals for hepatitis C have had an extraordinary impact on a major public health problem in just a few years, but they are not a panacea. Moreover, determining whether someone has hepatitis C is trivial compared to determining whether they have LOAD or what their future risk for developing it might be. Given the difficulties of AD diagnosis, its propensity to be confused with other diseases of aging, and uncertainty with regard to how to interpret biomarkers and other surrogates (e.g., cognitive testing), finding a definition of disease modification (and assigning a value to it) for LOAD that is palatable to patients, regulators, payers, health technology assessment bodies, service providers, caregivers, and the biopharmaceutical industry, represents a monumental challenge (Gallacher et al. 2019).

One recent proposal for assessing the value of gene therapies calls for a checklist that can be tailored to specific treatments for different diseases (Drummond et al. 2019). The authors divide their sample checklist into three headings: clinical effectiveness; elements of value; and “other considerations.” The clinical effectiveness section asks, among other things, whether the disease being treated with gene therapy represents an area of unmet medical need for a life-shortening condition, and about the nature of the clinical trials used to establish the treatment’s efficacy. The section assessing elements of value might consider the economic benefits of mitigating (or preventing) the condition and the direct and indirect benefits to, for example, caregivers, insurers, and science (via so-called “scientific spillover”). Finally, other considerations might include how the discount rate and cost-benefit analysis of the therapy are assessed and what assumptions underlie such calculations (Drummond et al. 2019).

For LOAD, some checklist items are apt to be straightforward: the disease clearly represents an unmet medical need that shortens life and imposes a massive financial burden on patients, families and institutions—indeed, on all of us. Other factors will require nuance and an ongoing iterative approach. For example, as discussed, AD diagnosis is currently imperfect, which has a bearing on clinical trial enrollment and outcomes and which, in turn, can impinge upon assessments of clinical effectiveness and value. For their part, considerations of elements of value should include both the sheer size of the LOAD patient population and its sharp growth curve, but also distinguish a DMT for LOAD from gene therapies for SMA and other pediatric diseases that are orders of magnitude less common and have the potential to prolong a very young life by many decades. For example, the per-capita value of LOAD patients returning to work following a DMT is likely to be a small fraction of the per-capita value of, say, a curative treatment for diseases that affect a younger population (e.g., hepatitis C, SMA, hemophilia). It is worth nothing that the Institute for Clinical and Economic Review has determined that, based on its substantial benefits to presymptomatic infantile-onset SMA patients, all eligible SMA patients could be treated with the gene therapy Zolgensma (onasemnogene abeparvovec, Novartis AG/AveXis) at a price of $2 million per one-time dose without exceeding ICER’s budget impact threshold (ICER 2019). Zolgensma’s 2020 list price was $2.1 million in the US (Miller 2020).

## All-you-can-eat or layaway? Picking up the tab for a DMT

Whatever the value calculations and even with the marketing of Zolgensma as a modestly instructive example, it would be presumptuous to think that we know exactly what a gene-editing-based DMT for AD might cost. But given the price tags for FDA-approved nucleic-acid-based treatments for other—admittedly rarer—diseases, it seems reasonable to assume that the costs will be quite high. Pharmaceutical companies will want to charge high prices to recoup the expenses they incur for large clinical trials involving thousands of LOAD patients. They will cite manufacturing and ongoing research and development costs. They will almost certainly amass intellectual property that affords one or, at most, a few companies patent exclusivity and allows them to charge monopolistic prices for a period of many years. They will also likely claim that finally delivering a meaningful clinical benefit to patients with a disease as costly, burdensome and debilitating as LOAD entitles them to charge a substantial premium.

Even under optimal conditions that partly distributes the financial obligations among millions of patients and families along with multiple insurers, the massive financial hit prompted by so many being treated with a drug costing tens or even hundreds of thousands of dollars would obviously not be workable for any healthcare system. But what about the cost savings brought about by a DMT? Unfortunately, it’s not clear when or to what extent they would be realized. We’ve already mentioned the reduced likelihood of healthy LOAD patients of retirement age returning to work. And as Wimo and colleagues discovered in modeling a DMT for prodromal/presymptomatic AD (albeit not a one-time gene therapy but rather an ongoing medication), preventing onset of AD would likely bring about little savings in the initial years and moreover, doing so would—we would hope—prolong survival and therefore lead to an uptick in the costs associated with normal aging for a population that includes many who would have otherwise died (Skoldunger et al. 2013; Wimo 2018).

Given LOAD’s disproportionate effect on the elderly, an effective DMT may well require innovative payment models in order to succeed in a fragmented US healthcare market. A number of such models have emerged in recent years; here, in broad terms, we will consider two.

One innovative approach is the so-called “Netflix model,” a shorthand for any subscription-based payment plan (Trusheim et al. 2018). Arguably the most salient real-world example of this approach has been for direct-acting antivirals for the treatment of hepatitis C. In June 2019, the Louisiana health department announced that, for a flat fee of $58 million per year, it would receive unlimited access to Gilead subsidiary Asegua’s hepatitis C drugs for 5 years, thereby allowing the state to treat as many Louisianans as it can, rather than having its budget constrained by enormous per-patient subscription prices ($20,000 to $30,000 each) (Deslatte 2019).^2^ If Louisiana’s cost for hep C drugs exceeds the fixed expenditure cap imposed by Asegua, then treatment after that is free for Louisiana patients. Under this arrangement, more Louisiana residents received hepatitis C treatment in the first 11 weeks of the new pact than in the entire prior fiscal year (Packer-Tursman 2019). That said, without true cost certainty for drug manufacturers, other states will likely find it difficult to implement subscription models (Pipes 2019).

Clearly, this model has promise. But even with cost certainty, in the case of LOAD we are again confronted by complicating factors. One that we’ve already mentioned is scale: thus far, gene therapies at or near the end of the pipeline have been targeted at rare or even ultra-rare diseases. In the Louisiana case, the stated goal was to get access to treatment for 31,000 Medicaid hepatitis C patients by 2024 (Deslatte 2019). How will the calculus change when the patient population covered by the subscription model is orders of magnitude larger and, because of the shape of the American demographic curve, growing? Another challenge is manufacturing costs. While production costs for small-molecule drugs tend to be low (Trusheim et al. 2018), gene-editing manufacturing costs—including quality control, regulatory provisions, and inpatient infusions—are certain to be substantially higher (Abou-El-Enein et al. 2014). Finally, the gene-editing space comes with formidable barriers to entry, including the aforementioned manufacturing costs as well as vigorous enforcement of intellectual property rights. Thus, even though LOAD represents a huge potential market that, by definition, will be unfettered by orphan drug exclusivity reserved for rare diseases, the competitive bidding process envisioned by Bach and colleagues might not materialize for many years after initial approval of a DMT (Trusheim et al. 2018).

A second model is exemplified by Bluebird Bio’s recently approved (in Europe) one-time gene therapy Zynteglo (“LentiGlobin”; autologous CD34+ cells encoding βA-T87Q-globin gene) for transfusion-dependent β-thalassaemia (Thompson et al. 2018). The therapy’s list price is $1.8 million. Under Bluebird’s sequential payment plan, upon the first (and presumably only) infusion of a thalassemia patient with Zynteglo, Bluebird would receive $355,000. The remaining $1.445 million will be divided among four equal payments over the subsequent 4 years, assuming that each year the patient continues to benefit, i.e., each year they do not require blood transfusions to mitigate their disease. But if a patient after, say, the first year, does require a transfusion, then Bluebird will lose that payment. If the patient’s condition then improves the following year such that they do not need a transfusion, then Bluebird would get paid (Feuerstein 2019). As of early 2020, multiple European insurers had reportedly signed onto the sequential payment arrangement (Tong 2020); as of August 2020, the company anticipated treating European thalassemia patients by the end of the year.^3^

The appeal of Bluebird’s plan is that it is outcomes based and does indeed give the company “skin in the game,” putting as much as 80% of the company’s revenue at risk (Feuerstein 2019). For a DMT for LOAD, such a plan could amortize the huge costs and thereby lessen their pain to payers. The challenges again come back to some of the distinguishing characteristics of LOAD. First, Bluebird chose a binary outcome to determine payment—a post-therapy thalassemia patient will either need a transfusion or not. What would an analogous measure be in LOAD? One could imagine improved performance on cognitive tests above a certain threshold, which would likely be the metric of highest concern to caregivers. But one might argue that a more objective measure—imaging for tau and beta amyloid, for example—would offer a more robust and reproducible test that was less subject to gaming. Second, the advanced age of onset in AD means that a significant fraction of patients will not survive their payment plans. So what happens if a patient—even an optimal responder—dies before their treatment is paid for? Presumably there would have to be some discount built into such a plan based on actuarial projections. And even under optimal circumstances, the lifespan of the typical LOAD patient is still going to be decades shorter than that of the typical thalassemia patient. Finally, any longterm payment plan must account for so-called “beneficiary churn,” that is, the mostly American phenomenon of people switching insurers that is strongly associated with physician turnover (Hsu et al. 2017). As the American Society of Gene and Cell Therapy has noted, such movement has the potential to sow confusion over which payer is responsible for covering the costs of a patient’s expensive gene therapy (Salzman et al. 2018).

## Conclusions and a potential path forward

The availability of a durably efficacious gene-editing-based DMT for LOAD would represent a remarkable triumph for public health and bring profound benefit to millions of Alzheimer’s disease patients and their caregivers. The most daunting hurdle will be to ensure that such a blessing does not become a curse. LOAD poses distinct challenges by virtue of its difficult differential diagnosis, its late onset, and its overwhelming numbers of patients for which, in these early days of gene therapy and gene editing, there is no precedent—we have no guarantee that the current frameworks for delivering access to gene therapies for much rarer diseases can be made to work at scale. To prepare for an effective DMT, we must therefore bring stakeholders together to:

- *Mandate early stratification of large patient populations*. By identifying younger people with strong family histories of AD, aggregate high genetic risk scores, telltale imaging and biomarkers, and perhaps cognitive and/or lifestyle vulnerabilities, it should be possible to assemble large Phase I-ready cohorts in relatively short order. It will be critical to analyze the costs and benefits of deep phenotyping and multifarious diagnostics versus lower-cost, higher-volume screening of the broader population. Millions of us will develop LOAD; relatively few of us will develop monogenic metabolic disorders.
- *Reimagine service delivery*. The Edinburgh Consensus concluded that a DMT will require the current symptomatic and palliative focus of dementia services to be reconfigured to identify and accommodate presymptomatic individuals (Ritchie et al. 2017). What incentives might be put into place to encourage trainees to develop such services and help evolve specialties like geriatrics, neurology, psychiatry, and other LOAD-adjacent fields? To what extent could the demands of diagnostics and clinical trials be shared with primary caregivers and allied health professionals? How can such a reconfiguration be implemented without neglecting advanced-stage LOAD patients unable to benefit from a DMT aimed at presymptomatic patients? These sorts of questions will require years of planning in the realms of medical education, capital infrastructure, clinic organization, reimbursement, and more.
- *Collect prospective cost data.* The true value of a DMT can only be accurately assessed if we have large-scale granular data for the cost of care for advanced-stage LOAD patients, early- and prodromal-stage patients, and cognitively healthy aging adults. The notorious lack of transparency in US healthcare makes this problem particularly vexing vis-à-vis countries with single-payer systems.
- *Consider both divergent and convergent financial interests.* Without consensus and widespread stakeholder buy-in, access to DMTs for common diseases runs the risk of exacerbating existing health disparities and perhaps fueling new ones. The sooner that patients, caregivers, payers, providers, policy analysts, and pharmaceutical companies begin to consider mutually beneficial ways to improve access to a DMT, the more likely they will be to develop innovative ways to address the challenges posed by an expensive drug needed by millions.

It’s worth reiterating that the need for collective action by a large group of stakeholders is a direct function of the magnitude of the challenge of safe, equitable, and sustainable development *and* delivery of a DMT for LOAD. First, we minimize the scientific challenges of creating a DMT for LOAD at our peril—decades of work and dozens if not hundreds of failed drug trials speak to this in the starkest of terms. But our main goal here has been to consider what happens when those challenges are overcome. The object lessons we discuss (SMA, hepatitis C) and others that we don’t (e.g., cancer) are all imperfect analogues, whether because of the nature of LOAD epidemiology (i.e., it is not rare and tends to strike late), its diagnosis, its onset and progression, its heterogeneity, and/or its peculiar demands on caregivers and loved ones. All of these complicating factors have led to nucleic acid-based DMTs for monogenic rare diseases reaching the market well before similar therapies for diseases like LOAD. Thus, the certainty with which we can extrapolate from the rare to the common is limited by both the sharp distinctions between the two and our limited experience with the latter. For Alzheimer’s disease, the scientific and societal hurdles are both enormous and closely related to one another. Our hope is that we can start planning for success sooner rather than later.

## Data Availability

Not applicable (review article).

## Abbreviations

AD: Alzheimer’s disease
DMT: Disease-modifying therapy
FDA: US Food and Drug Administration
ICER: Institute for Clinical and Economic Review
LOAD: Late-onset Alzheimer’s disease
SMA: Spinal muscular atrophy

## Footnotes

1 https://www.cdc.gov/nchhstp/newsroom/2018/hepatitis-c-prevalence-estimates.html

2 https://www.documentcloud.org/documents/6386058-Louisiana-Asegua-Therapeutics-Contract-Hepatitis.html

3 https://finance.yahoo.com/news/bluebird-bio-reports-second-quarter-201000429.html

## References

[CR1] Alzheimer's Association. 2020. 2020 Alzheimer's disease facts and figures. Alzheimer's and Dementia. 16(3):391– 460. 10.1002/alz.12068 10.1002/alz.12068 32157811

[CR2] Abou-El-Enein M, Bauer G, Reinke P (2014). The business case for cell and gene therapies. Nature Biotechnology..

[CR3] Al-Zaidy S, Pickard AS, Kotha K (2019). Health outcomes in spinal muscular atrophy type 1 following AVXS-101 gene replacement therapy. Pediatric Pulmonology.

[CR4] Barker RA, Gotz M, Parmar M (2018). New approaches for brain repair-from rescue to reprogramming. Nature..

[CR5] Baumert TF, Berg T, Lim JK (2019). Status of direct-acting antiviral therapy for Hepatitis C virus infection and remaining challenges. Gastroenterology..

[CR6] Begley S (2019). The maddening saga of how an Alzheimer's 'cabal' thwarted progress toward a cure for decades.

[CR7] Brown, E.E., S. Kumar, T.K. Rajji, et al. 2020. Anticipating and mitigating the impact of the COVID-19 pandemic on Alzheimer's disease and related dementias. *The American Journal of Geriatric Psychiatry*. 10.1016/j.jagp.2020.04.010. 10.1016/j.jagp.2020.04.010 PMC7165101 32331845

[CR8] Bustos FJ, Ampuero E, Jury N (2017). Epigenetic editing of the Dlg4/PSD95 gene improves cognition in aged and Alzheimer's disease mice. Brain..

[CR9] Cohen J (2013). Pharmaceuticals. Advocates protest the cost of a hepatitis C cure. Science..

[CR10] Corneliussen-James CD (2015). IN02 metavivor: Fighting prejudice. The Breast..

[CR11] Cota-Coronado A, Diaz-Martinez NF, Padilla-Camberos E (2019). Editing the central nervous system through CRISPR/Cas9 systems. Frontiers in Molecular Neuroscience.

[CR12] Dall TM, Storm MV, Chakrabarti R (2013). Supply and demand analysis of the current and future US neurology workforce. Neurology..

[CR13] Darrow JJ (2019). Luxturna: FDA documents reveal the value of a costly gene therapy. Drug Discovery Today.

[CR14] Deslatte, M. 2019. Louisiana reaches 'Netflix-model' deal to tackle hepatitis C. New York: Associated Press. https://apnews.com/bc074b5c06024926a5c58163de8bab9d. Accessed 11 Sept 2020.

[CR15] Douglass CH, Pedrana A, Lazarus JV (2018). Pathways to ensure universal and affordable access to hepatitis C treatment. BMC Medicine.

[CR16] Drummond MF, Neumann PJ, Sullivan SD (2019). Analytic considerations in applying a general economic evaluation reference case to gene therapy. Value in Health.

[CR17] Dufouil C, Glymour MM (2018). Prediction to prevention in Alzheimer's disease and dementia. Lancet Neurology.

[CR18] Dujardin S, Commins C, Lathuiliere A (2020). Tau molecular diversity contributes to clinical heterogeneity in Alzheimer's disease. Nature Medicine.

[CR19] El-Hayek, Y.H., R.E. Wiley, C.P. Khoury, et al. 2019. Tip of the iceberg: Assessing the global socioeconomic costs of Alzheimer's disease and related dementias and strategic implications for stakeholders. *Journal of Alzheimer's Disease*. 10.3233/JAD-190426. 10.3233/JAD-190426 PMC6700654 31256142

[CR20] Feuerstein A (2019). Bluebird's gene therapy for a rare blood disease will cost $1.8 million. Cue the pricing debate.

[CR21] Gallacher J, de Reydet de Vulpillieres F, Amzal B (2019). Challenges for optimizing real-world evidence in Alzheimer's disease: The ROADMAP project. Journal of Alzheimer's Disease.

[CR22] Garde D (2019). Experimental gene therapy for hemophilia, developed by Pfizer and Sangamo, shows early promise.

[CR23] Geifman N, Kennedy RE, Schneider LS (2018). Data-driven identification of endophenotypes of Alzheimer's disease progression: Implications for clinical trials and therapeutic interventions. Alzheimer's Research & Therapy.

[CR24] Giau VV, Lee H, Shim KH (2018). Genome-editing applications of CRISPR-Cas9 to promote in vitro studies of Alzheimer's disease. Clinical Interventions in Aging.

[CR25] Hampel H, Vergallo A, Giorgi FS (2018). Precision medicine and drug development in Alzheimer's disease: The importance of sexual dimorphism and patient stratification. Frontiers in Neuroendocrinology.

[CR26] Hersi M, Irvine B, Gupta P (2017). Risk factors associated with the onset and progression of Alzheimer's disease: A systematic review of the evidence. Neurotoxicology..

[CR27] Hsu J, Vogeli C, Price M (2017). Substantial physician turnover and beneficiary ‘churn’in a large Medicare Pioneer ACO. Health Affairs..

[CR28] Huang YM, Shen J, Zhao HL (2019). Major clinical trials failed the amyloid hypothesis of Alzheimer's disease. Journal of the American Geriatrics Society.

[CR29] ICER (2019). Spinraza and Zolgensma for Spinal Muscular Atrophy: Effectiveness and Value. Final Evidence Report. (ICER: Institute for Clinical and Economic Review).

[CR30] Iqubal, A., M.K. Iqubal, A. Khan, et al. 2020. Gene therapy, a novel therapeutic tool for neurological disorders: Current progress, challenges and future prospective. *Current Gene Therapy*. 10.2174/1566523220999200716111502. 10.2174/1566523220999200716111502 32674730

[CR31] Keshavan M (2019). On Alzheimer’s, scientists head back to the drawing board — and once-shunned ideas get an audience.

[CR32] Khan TK, Alkon DL (2015). Peripheral biomarkers of Alzheimer's disease. Journal of Alzheimer's Disease.

[CR33] Ko, Y., and S.M. Chye. 2020. Lifestyle intervention to prevent Alzheimer's disease. *Reviews in the Neurosciences*. 10.1515/revneuro-2020-0072. 10.1515/revneuro-2020-0072 32804681

[CR34] Lange C, Suppa P, Pietrzyk U (2018). Prediction of Alzheimer's dementia in patients with amnestic mild cognitive impairment in clinical routine: Incremental value of biomarkers of neurodegeneration and brain amyloidosis added stepwise to cognitive status. Journal of Alzheimer's Disease.

[CR35] Loftus, P. 2014. Senate committee is investigating pricing of hepatitis C drug. *The Wall Street Journal.* 11. https://www.wsj.com/articles/senate-finance-committee-is-investigating-pricing-of-hepatitis-c-drug-1405109206. Accessed 11 Sept 2020.

[CR36] Lombardi G, Crescioli G, Cavedo E (2020). Structural magnetic resonance imaging for the early diagnosis of dementia due to Alzheimer's disease in people with mild cognitive impairment. Cochrane Database of Systematic Reviews.

[CR37] Marchione, M. 2020. Scientists get closer to blood test for Alzheimer’s disease. New York: Associated Press. https://apnews.com/4d9f28d2051889e8326f1e9ca5ec5676. Accessed 11 Sept 2020.

[CR38] Miller J (2020). Novartis wins conditional EU approval for gene therapy Zolgensma.

[CR39] Packer-Tursman J. 2019. Louisiana’s Hepatitis C 'Netflix' Model Sees Positive Results. Washington: AIS Health. https://aishealth.com/drug-benefits/louisianas-hepatitis-c-netflix-model-sees-positive-results/. Accessed 11 Sept 2020.

[CR40] Pipes S. 2019. Louisiana’s Tries Hard, But Federal Obstacles Cause Hepatitis C Plan to Fall Short. New Jersey: Forbes. https://www.forbes.com/sites/sallypipes/2019/08/07/louisiana-tries-hard-but-federal-obstacles-cause-hepatitis-c-plan-to-fall-short/#672603b36ec7. Accessed 11 Sept 2020.

[CR41] Pitkälä KH, Martin FC, Maggi S (2018). Status of Geriatrics in 22 Countries. The Journal of Nutrition, Health & Aging.

[CR42] Prendecki, M., M. Kowalska, E. Toton, et al. 2020. Genetic editing and pharmacogenetics in current and future therapy of neurocognitive disorders. *Current Alzheimer Research*. 10.2174/1567205017666200422152440. 10.2174/1567205017666200422152440 32321403

[CR43] Rabin, J.S., H. Klein, D.R. Kirn, et al. 2019. Associations of physical activity and β-amyloid with longitudinal cognition and neurodegeneration in clinically normal older AdultsAssociations of physical activity and β-amyloid with cognition and Neurodegeneration in clinically normal older AdultsAssociations of physical activity and β-amyloid with cognition and neurodegeneration in clinically normal older adults. *JAMA Neurology.*10.1001/jamaneurol.2019.1879. 10.1001/jamaneurol.2019.1879 PMC6635892 31312836

[CR44] Raikwar SP, Thangavel R, Dubova I (2018). Neuro-immuno-gene- and genome-editing-therapy for Alzheimer's disease: Are we there yet?. Journal of Alzheimer's Disease.

[CR45] Ricciarelli R, Fedele E (2017). The amyloid cascade hypothesis in Alzheimer's disease: It's time to change our mind. Current Neuropharmacology.

[CR46] Riedel BC, Daianu M, Ver Steeg G (2018). Uncovering biologically coherent peripheral signatures of health and risk for Alzheimer's disease in the aging brain. Frontiers in Aging Neuroscience.

[CR47] Ritchie CW, Russ TC, Banerjee S (2017). The Edinburgh consensus: Preparing for the advent of disease-modifying therapies for Alzheimer's disease. Alzheimer's Research & Therapy.

[CR48] Salzman, R., F. Cook, T. Hunt, et al. 2018. Addressing the Value of Gene Therapy and Enhancing Patient Access to Transformative Treatments. *Molecular Therapy*. 26(12):2717–2726. 10.1016/j.ymthe.2018.10.017. Epub 2018 Oct 30. 10.1016/j.ymthe.2018.10.017 PMC6277509 30414722

[CR49] Skoldunger A, Johnell K, Winblad B (2013). Mortality and treatment costs have a great impact on the cost-effectiveness of disease modifying treatment in Alzheimer's disease-a simulation study. Current Alzheimer Research..

[CR50] Thompson AA, Walters MC, Kwiatkowski J (2018). Gene therapy in patients with transfusion-dependent beta-thalassemia. The New England Journal of Medicine.

[CR51] Tong A (2020). Celgene exec jumps to head bluebird bio ops in Europe, where its $1.8M gene therapy Zynteglo is now available.

[CR52] Trusheim MR, Cassidy WM, Bach PB (2018). Alternative state-level financing for hepatitis C treatment-the "Netflix model". JAMA..

[CR53] Turner, R.S., M.L. Hebron, A. Lawler, et al. 2020. Nilotinib effects on safety, tolerability, and biomarkers in Alzheimer's disease. *Annals of Neurology*. 10.1002/ana.25775. 10.1002/ana.25775 PMC7383852 32468646

[CR54] van der Lee SJ, Wolters FJ, Ikram MK (2018). The effect of APOE and other common genetic variants on the onset of Alzheimer's disease and dementia: A community-based cohort study. Lancet Neurology.

[CR55] Voretigene neparvovec-rzyl (Luxturna) for inherited retinal dystrophy. 2018. *The Medical Letter on Drugs and Therapeutics* 60:53–55. https://pubmed.ncbi.nlm.nih.gov/29635265/29635265

[CR56] Watt AD, Jenkins NL, McColl G (2019). Ethical issues in the treatment of late-stage Alzheimer's disease. Journal of Alzheimer's Disease.

[CR57] Wimo A (2018). The end of the beginning of the Alzheimer's disease nightmare: A Devil's Advocate's view. Journal of Alzheimer's Disease.

